# Regional anaesthesia research – where to now?

**DOI:** 10.1177/20494637221091139

**Published:** 2022-04-08

**Authors:** Rachel J Kearns, Jonathan Womack, Alan JR Macfarlane

**Affiliations:** 1 Department of Anaesthesia, 59736 Glasgow Royal Infirmary, NHS Greater Glasgow and Clyde, Glasgow, UK; 2 School of Medicine, 3526 University of Glasgow, Glasgow, UK; 3 Department of Anaesthesia, 105563 Royal Victoria, Infirmary, Newcastle Upon Tyne Hospitals NHS Foundation Trust, Newcastle Upon Tyne, UK

Regional anaesthesia (RA) is in the midst of a renaissance. The advent of ultrasound guidance has not only increased efficacy and improved safety, but has also led to a plethora of novel techniques. Fascial plane blocks in particular are appearing in the literature at an almost exponential rate.^1,2^ The ongoing SARS-CoV-2 pandemic has highlighted the potential benefits of RA,^3–6^ and the updated RCoA 2021 curriculum for anaesthetists in training recognises RA as an area of increasing focus and attention.^7^ Nevertheless, RA remains underutilised, most likely due to a combination of many anaesthetists lacking the technical proficiency, other healthcare professionals having reservations about its use, and a lack of definitive evidence of superiority compared to alternative techniques in some circumstances.^8–10^ Evidence of benefit is accumulating however, and RA is evolving from an area dominated by enthusiasts to an expected component of a modern anaesthetist’s skillset. It is important that we strive to deliver training to match this objective.^7^ Whilst the burgeoning interest and enthusiasm for RA is welcome, it is important that the patient alongside the multi-disciplinary team, is not only involved but at the forefront of all decisions surrounding their management. This is true not only for clinical care, but for the research that informs and drives it.

The RA-UK regional anaesthesia research network was set up in 2021, and a census of current work has revealed an encouraging breadth and depth of studies amongst the UK RA research community.^11^ Large scale multi-centre NIHR funded trials are underway,^12,13^ and a new swathe of studies are exploring the use of augmented reality and artificial intelligence technologies to enhance RA training and performance.^14,15^ A number of recently published international collaborations will help guide teaching, education, and clinical practice, improving the potential for comparative research and advancing our understanding of the clinical applications of RA.^16–20^ Whilst generation of new evidence is vital, it is equally important to ensure that important research findings are translated in to clinical practice. The production of clinical guidelines has long bridged the gap between evidence synthesis and implementation in practice, and the role of local “champions” is recognised as a powerful influence for instituting change.^21^ As suggested in Royal College of Anaesthetists' guidance, there are local leads for many aspects of anaesthetic care.^22^ This is not the case for RA, and it may be there is a role for either departmental RA ‘leads’, or a national link network to coordinate RA training and implementation locally.^23,24^

What then of future research in regional anaesthesia? As the number of blocks being described continues to rise, there is a growing appreciation that *“new”* or *“more complex”* does not always translate to *“better”*.^25^ It is becoming increasingly accepted that we must exercise caution in adopting new blocks without a solid evidence base, particularly where a well-researched, validated, and simpler alternative exists.^25^ From a clinical practice viewpoint, the “Plan A” block concept focuses on promoting basic nerve blocks that are most likely to add value to patient care.^16^ However, the question of how we define *“value”*, or in other words, what outcomes are the most relevant and important, remains incompletely understood.^26^ We must strive to answer this question this if we are to maximise the reach and impact of RA research.

Contemporary RA trials seek not only to examine the more traditional outcomes of pain (both acute and chronic), opioid related side-effects, and patient satisfaction but aim to refine these to be more specific to RA interventions whilst addressing confounding factors such as socioeconomic disadvantage, cultural differences, prior experience, and expectations.^25^ The British Pain Society and Faculty of Pain Medicine have produced guidance advising on the importance of investigating the multi-facets of important study outcomes such as acute and chronic pain (e.g., pain interference, physical and emotional functioning, and quality of life) to provide more tangible information to patients and practitioners.^27^ For example, in a patient who has sustained rib fractures, traditional visual analogue pain scores and opioid consumption may be difficult to interpret in the context of other injuries, and quality of recovery scores are generally validated in the post-operative rather than polytrauma setting.^28,29^ Functional measures of analgesia and recovery present a more important endpoint, but there is currently little evidence to suggest what patients with rib fractures consider a successful analgesic intervention for the pain they experience. The inclusion of functional outcomes such as mobility, strength, quality of recovery, and quality of life (with an emphasis on the minimal clinically relevant difference we should be testing), is widely recognised as being an important aspect of trial design.^30–33^ The Core Outcome Measures in Effectiveness Trials (COMET) programme provides invaluable guidance in standardising study outcomes, but despite much progress, many of the more patient-centred and functional outcomes require further study and questions remain. For example, can RA affect longer-term surgical, functional and patient-centred outcomes? Can RA affect the recurrence of malignancy, and is it realistic to expect such a short-term peri-operative intervention to do so? Should we not simply be satisfied that RA significantly improves patient comfort and provides other short-term benefits in the acute post-operative phase, or should we expect more? The next era of RA research will potentially answer some of these questions.

It is now widely accepted by researchers, funders, and journals that it is essential to establish the views of patients and caregivers, as well as healthcare professionals, when considering what we might research to improve their quality of life.^34^ For research to have true impact, it must not only include, but be co-designed by the people it seeks to help. The RA-UK research network, alongside its partner organisations, has launched a UK research priority setting exercise seeking to determine the top ten RA research questions. This will include a wide range of participants from a diverse range of professional and specialty backgrounds. Most importantly, this exercise will include patients and caregivers, both as steering group members and as survey respondents. The survey will be performed electronically in simple, accessible language and will ask open questions on what is important to research in RA. Answers will be collated, summarised and grouped into themed questions, and a further survey performed to prioritise these questions. The UK RA research priorities will be finalised by panel discussion including patients and representatives from all disciplines. A second process surveying regional anaesthetists worldwide will also be used to generate a set of global RA research priorities. In doing this work, we hope to galvanise both researchers and funders on what RA research should be prioritised in order to drive the most significant and impactful benefits for patient care. We would encourage everyone to have their say.
